# SEMA3C Supports Pancreatic Cancer Progression by Regulating the Autophagy Process and Tumor Immune Microenvironment

**DOI:** 10.3389/fonc.2022.890154

**Published:** 2022-06-16

**Authors:** Dalin Zhang, Aaron Lindstrom, Edward J Kim, Chang-il Hwang, Madison Lee Hall, Tzu-Yin Lin, Yuanpei Li

**Affiliations:** ^1^ Department of Biochemistry and Molecular Medicine, UC Davis Comprehensive Cancer Center, University of California, Davis, Sacramento, CA, United States; ^2^ Division of Hematology and Oncology, Department of Internal Medicine, University of California, Davis, Sacramento, CA, United States; ^3^ Department of Microbiology and Molecular Genetics, University of California, Davis, Davis, CA, United States

**Keywords:** TCGA, SEMA3C, KRAS G12D, Pancreatic Cancer, Autophagy, Tumor Immune Microenvironment

## Abstract

To date, driver genes for pancreatic cancer treatment are difficult to pursue therapeutically. Targeting mutated KRAS, the most renowned driver gene in pancreatic cancer, is an active area of study. We discovered a gene named SEMA3C was highly expressed in pancreatic cancer cell lines and patients with a G12D mutation in KRAS. High expression of SEMA3C in patients was significantly associated with the decreased survival of pancreatic cancer patients based on the TCGA database. In pancreatic cancer cells, SEMA3C knockdown or inhibition exhibited growth/colony inhibition and cell cycle arrest. In addition, SEMA3C inhibition sensitized KRAS or MEK1/2 inhibition in pancreatic cancer cells. Overexpression of SEMA3C resulted in the induction of autophagy, whereas depletion of SEMA3C compromised induction of autophagy. SEMA3C modified the PD-L1 expression in tumor and immune cells and is correlated with the M2-like macrophage marker ARG1/CD163 expression, which could reshape the tumor microenvironment. Inhibition of SEMA3C decreased tumor formation in the xenograft model *in vivo*. Taken together, our data suggest that SEMA3C plays a substantial role in promoting cancer cell survival by regulating the autophagy process and impacting the tumor environment immune response. SEMA3C can be used as a novel target or marker with therapeutic or diagnostic potential in pancreatic cancer especially in tumors harboring the specific KRAS G12D mutation.

## Introduction

Pancreatic cancer is the fourth highest cause of cancer deaths in men and women in the United States. It is predicted to be the second leading cause of cancer-related death by 2030 (1). The median survival is less than a year with the most aggressive type of pancreatic cancer. Despite advances in targeted therapies for many other cancers, the only option for treating the majority of pancreatic cancers remains chemotherapy (2). Overall, pancreatic tumors have a limited response to cytotoxic drugs, which contributes to the persistently poor prognosis for pancreatic cancer patients. Recently the 5-year survival rate for all stages of pancreatic cancer is 11%, and patients who are diagnosed at a late stage have a 5-year survival rate of only 3% (3). Gemcitabine is a standard treatment for pancreatic cancer, with a history of more than 20 years, but no longer a standard monotherapy treatment except in selected cases of frail patients who cannot tolerate the more aggressive regimens like Folfirinox and Gem/nab-paclitaxel (4). To date, there are few therapeutically targetable driver genes for pancreatic cancer. A limited number of molecularly targeted drugs, such as *PARP*, *BRCA*, and *NTRK* inhibitors, are in the clinical trial stage for pancreatic cancer (5).

More than 90% of pancreatic tumors harbor a KRAS mutation, which is considered an early driver mutation essential in the initiation of pancreatic carcinogenesis (6). Substantial evidence shows that mutated KRAS is essential for PDAC growth, but the K*RAS* protein has been generally considered undruggable for many years because it does not have a suitable pocket for the compound binding with high affinity. Recently, sotorasib (AMG510), a compound directly targeted to KRAS with the G12C mutation, was shown to cause the regression of KRAS G12C lung carcinoma. AMG510 is the first KRAS (G12C) inhibitor in clinical development and treatment and was recently approved by FDA for the lung cancer therapy (7). Nonetheless, AMG510 was not effective on the KRAS G12D mutation types because of the changed binding pocket (8) and the tumor will quickly acquire resistance to KRAS G12C inhibition. Therefore, new therapeutic strategies are needed to develop and overcome this drug resistance in patients with cancer (9). Furthermore, evidence shows that the origins and genetic interactions of KRAS mutations are allele and tissue specific (10). For instance, increased AKT phosphorylation was observed in KRAS G12D expressing cell lines, whereas increased RAL-GTP was detected in G12C cell lines (11). In colon cancer, DCLK1 and the MET are upregulated in KRAS G12 mutant expressing KRAS cells, whereas ZO-2 is upregulated in KRAS G13D (12). These studies illustrate that individual KRAS mutants can mediate different signaling pathways and require unique pharmacological targeting for different subtypes. Recently, efforts have centered on alternative strategies to inhibit the different KRAS mutation signaling pathway, such as STK33 (13), GATA2 (14), p38 (15), SLC7A11 (16),and PI3K (17). These proteins mediate different signaling pathways that support cancer progression and have been explored for the KRAS mutation dependent genes. Exploring new dependent genes or pathways for different *KRAS* mutation subtypes could lead to the identification of targetable vulnerabilities and the development of new treatments.

SEMA3C was reported as an oncogene and could support the tumor progression in pancreatic cancers. SEMA3C promotes tumor growth and metastasis in pancreatic cancer by activating the ERK 1/2 signaling pathway (18). In other tumors, SEMA3C is also reported to be an oncogene. For example, SEMA3C is one of the top 20 most frequently altered genes and its expression is markedly increased compared to astrocytoma tissues and this increase was significantly associated with the shorter overall survival of patients in glioblastomas (19, 20). In addition, high expression of SEMA3C is associated with poor prognosis and progression in prostate, breast, liver, gastric, pancreatic, and lung cancer (21). SEMA3C also plays an important role in the maintenance of cancer stem-like cells. For example, SEMA3C is selectively expressed in Glioblastoma stem cells but not in their counterpart neural progenitor cells or non-stem tumor cells (22). Overexpression of SEMA3C in prostate cancer cell lines facilitates stem cell marker CD44 expression and tumor-sphere formation, suggesting a role for SEMA3C in maintaining prostate CSCs (23). SEMA3C also can take part in the migration or metastasis of cancer cells. SEMA3C is highly expressed in aggressive, highly metastatic Her2-positive breast cancer and SEMA3C depletion reduced cell migration (24, 25).

Here, we found that SEMA3C was highly expressed in pancreatic cancer and associated with the KRAS G12D mutation. Knockdown of SEMA3C expression significantly decreased tumor growth *in vitro* and *in vivo*. SEMA3C regulates the autophagy process and enhances the tumor immunosuppressive related genes expression of macrophage, thus targeting the SEMA3C is a promising target in pancreatic cancer therapy.

## Materials and Methods

### Reagents

Anti-ACTIN, Anti-ULK1, Anti-p-ULK1, Anti-p62, and Anti-LC3B were purchased from Cell Signaling Technology (Danvers, MA). The SEMA3C and GAPDH antibodies were purchased from Proteintech (Rosemont, IL). The Anti-KRAS antibody was from ABclonal (Woburn, MA). CellTiter-Glo^®^2.0 was purchased from Sigma-Aldrich (St. Louis, MO). ECL Chemiluminescence kit was obtained from National Diagnostics (Atlanta, Georgia). Recombinant Human SEMA3C protein was purchased from the R&D Systems (Minneapolis, MN). EasySep™ Mouse CD8+ T Cell Isolation Kit was from Stem Cell (Cambridge, MA). Human *KRAS* (G12D) Expression Lentivirus was from GenTarget Inc (San Diego, CA). *SEMA3C* plasmids were purchased from Sino Biological (Wayne, PA). The SEMA3C inhibitor, 3,5,4’-Tribromosalicylanilide (CAS: 87-10-5), was purchased from TCI chemicals (Portland, OR) (26).

### Bioinformatics Analysis

The GEO, TCGA, cBioPortal (27), GEPIA (28), Timer (29), KM plot (30), and Oncomine databases (31) were retrieved and applied for analysis according to the respective website guidelines.

### Cell Culture and Transfection

BxPC3, PANC1, PACA2, 3T3, and 293T cells were obtained from the American Tissue Culture Collection. KPC cells were kindly provided by Dr. Chang-il Hwang’s lab in UC Davis. The KPC-Luc cells were transfected by the lentivirus-luciferase and 2 mg/ml of puromycin was added for stable cell selection. A549 GFP-RFP-LC3B and Hela GFP-RFP-LC3B were kindly provided by Dr. Jeremy Chien’s lab in UC Davis. Cells were cultured in Dulbecco’s modified Eagle’s medium or RPMI 1640 (Gibco) supplemented with 10% fetal bovine serum (Sigma), 100 U/ml penicillin, and 100 mg/ml streptomycin (Sigma). Pancreatic cancer cells were transfected with target siRNA using a transfection reagent (GeneCopoeia) according to the manufacturer’s protocol. The *SEMA3C* siRNA sequences were 5′-CACCAUCCUUUAGACUACA-3′, *KRAS* siRNA sequences 5′-CUAUGGUCCUAGUAGGAAA-3′, and the Universal Negative Control siRNA was from Sigma-Aldrich.

### Immunoblotting Analysis

Immunoblotting analysis was performed as previously described (32). Cells were harvested, washed, and lysed in the RIPA buffer (Thermo fisher). Cell lysates were subjected to SDS-PAGE and transferred to PVDF membranes. PVDF membranes were then incubated with 5% (w/v) nonfat dry milk in TBST washing buffer (20 mM Tris-Cl, pH 7.6, 150 mM NaCl, and 0.1% Tween 20) for 1 h at 37°C to block nonspecific protein binding. Primary antibodies (1:1000) were diluted in the washing buffer containing 5% BSA and applied to the membranes overnight at 4°C. After extensive washing, the membranes were incubated with peroxidase-conjugated antibodies for 1 h at room temperature and washed again. Immunoreactive bands were visualized with the ECL Chemiluminescence kit.

### Growth Assay

Cells were seeded in 96-well plates at a density of 3000 cells/well. CellTiter-Glo^®^2.0 was used for detecting the cell numbers every 24 hours with a plate reader (Molecular Devices, San Jose, CA).

### Confocal Image

2×10^4^ A549 or Hela with GFP/RFP-LC3B cells were seeded in a culture dish with a glass-bottom plate overnight and treated with the SEMA3C inhibitor 25 µM 24 hours. Confocal laser scanning microscopy (LSM 800, ZEISS) was employed to monitor the patterns and changes of GFP and RFP fluorescence in cells.

### RT-PCR

Quantitative RT-PCR assays were performed as previously described (33). The total RNA was isolated using the TRIZOL reagent (Thermo Scientific, Waltham, MA) and the phenol-chloroform extraction method according to the manufacturer’s instructions. The cDNA was synthesized by the SuperScript reverse transcriptase (I Thermo Scientific, Waltham, MA) with 2 μg total RNA. qPCR was carried out with the SYBR Green PCR Master Mix (Thermo Fisher) on a CFX96 Real-Time PCR Detection System (Bio-Rad, Hercules, CA). GAPDH was used as a housekeeping gene to normalize the level of mRNA expression.

### Colony Assay

The cells were seeded into 6-well plates with a density of 2 × 10^3^ per well. On day 14, 0.005% crystal violet was added to make the colonies visible. Each assay was repeated three times.

### Conditional Medium

Cell conditioned media was collected from 90% confluent cultures following 48 hours of conditioning. To remove cellular debris, conditioned media underwent differential centrifugation before concentration (20X, 30kDa filter, Millipore) (34).

### T Cell Proliferation

CD8+ T cells were isolated from the spleen with the EasySep™ Mouse CD8+ T Cell Isolation Kit (Stem cell, MA). T cells then were activated by the anti-CD3 and anti-CD28 co-stimulation for 24 hours. 3T3 and KPC cells were transfected with the Vehicle or *SEMA3C* plasmid for 24 hours and then collected for co-culturing with the activated CD8+ T cells for 48 hours. Suspended live T cells numbers were counted by trypan blue staining.

### Animal Studies

All animal experiments were strictly in compliance with the guidelines of the Animal Use and Care Administrative Advisory Committee of the University of California, Davis (IACUC #20265). C57/BL6 mice were purchased from the Jackson Laboratory (Bar Harbor, ME). The subcutaneous tumor models were established by inoculating KPC cells (5 × 10^5^) into the flank of mice. After subcutaneous tumors reached approximately 50 mm^3^, SEMA3C inhibitor or saline were given by IP every other day (20 mg/kg) for a total of 7 doses (n=5/group). The body weight and volume of tumors were measured every three days. Animal spirit, hair coat, and behavior were also closely monitored. Animals were terminated two weeks after the therapy. Tumors and organs were then harvested and fixed for microscopic analysis. KPC cells transfected with vehicle and *SEMA3C* sgRNA 9# and 17# were subcutaneously injected into the C57/BL6 mice(n=6) and after the tumor reached around 50mm^3^, tumor volume was recorded every 3 days for 6 times.

### Statistical Analysis

All statistical analyses were conducted using GraphPad Prism (GraphPad Software, La Jolla, CA). Statistically significant differences between two independent groups were determined by Student’s *t*-test. The survival analysis was determined by the log-rank test. A value of P<0.05 was considered statistically significant.

## Results

### KRAS G12D Mutation Status and Expression Level are Associated With Poor Prognosis

**Figure 1:**
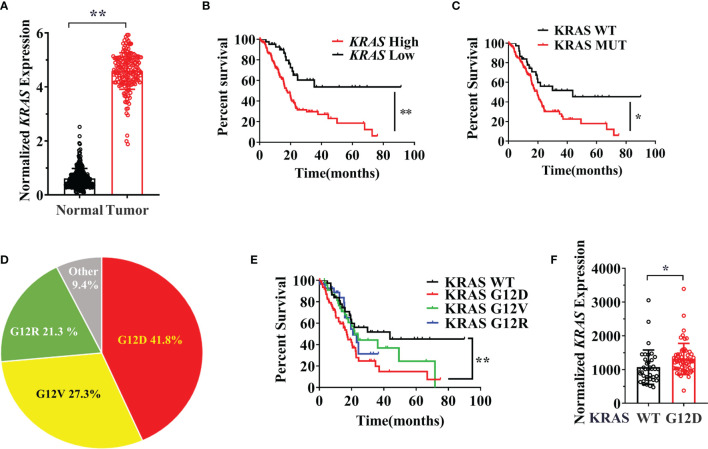
**(A)** The expression data are first log2(TPM+1) transformed for differential analysis and the log2FC is defined as median (Tumor) and median (TCGA normal + GTEx normal), data were collected and analyzed from GEPIA. **(B)** The overall survival of the KRAS expression. Data from KM plot, significance vs cutoff values between lower and upper quartiles of expression. **(C)** Survival analysis of the pancreatic cancer patients between the KRAS wildtype and KRAS mutation. **(D)**The proportion of different mutation types in pancreatic cancer. **(E)** Survival analysis of the pancreatic cancer patients among different KRAS mutation and wild type. **(F)** KRAS expression level in G12D mutation and wild type group. These data were from the cBioPortal database and http://firebrowse.org/. Data are representative of three independent experiments. T-tests or log-rank Test for the measurements between the two groups; *P < 0.05, **P < 0.01.

There is still a lack of clear understanding of the prognostic value of different KRAS mutation subtypes in pancreatic cancer. There are several studies on the KRAS mutation related prognosis and multiple studies stated that poor outcomes were identified in patients with KRAS G12D mutant pancreatic cancer (35, 36). However, other studies found no significant association between the G12D mutation and prognosis in pancreatic cancer patients (37, 38). Therefore, we first investigated the relationship between the KRAS mutation subtype and prognosis with the TCGA for pancreatic cancer patients and confirmed the prognosis benefit of the mutation type. As expected, KRAS was overexpressed in pancreatic cancer compared with non-tumor and KRAS expression caused a worse clinical outcome in pancreatic cancer (**Figures 1A, B**). Patients with KRAS mutated tumors were also significantly associated with a worse prognosis in overall survival of pancreatic cancer patients compared with KRAS WT tumors (**Figure 1C**). Interestingly, the ratio of KRAS mutation types varies in different cancers and the *KRAS* G12D mutation type made up the highest proportion of tumors harboring a KRAS mutation (**Figure 1D**). We next investigated whether the different subtypes of KRAS mutation in patients were associated with prognosis. We found that KRAS G12D mutation significantly correlated with worse survival when compared to KRAS wild type, KRAS G12R, and KRAS G12V mutations (**Figure 1E**). We also found that different KRAS mutations induced distinctly specific gene enrichment patterns and signaling pathway alterations in KRAS WT and Mutation cell lines. The 20 most different genes expression in KRAS-WT and MUTATION is different from different mutation types (**Figures S1, S2** and **Table S1**). Previous studies have shown that tumor KRAS gene expression levels are influenced by the KRAS mutational status and *KRAS* mutation leads to an increase in KRAS mRNA level, which can enhance the downstream signaling pathways (39). We confirmed this observation that the *KRAS* G12D mutation can lead to increased KRAS expression level in pancreatic cancer patient samples (**Figure 1F**).

### SEMA3C Expression is Associated With KRAS G12D Mutation and Highly Expressed in Pancreatic Cancers With a Poor Clinical Outcome

**Figure 2:**
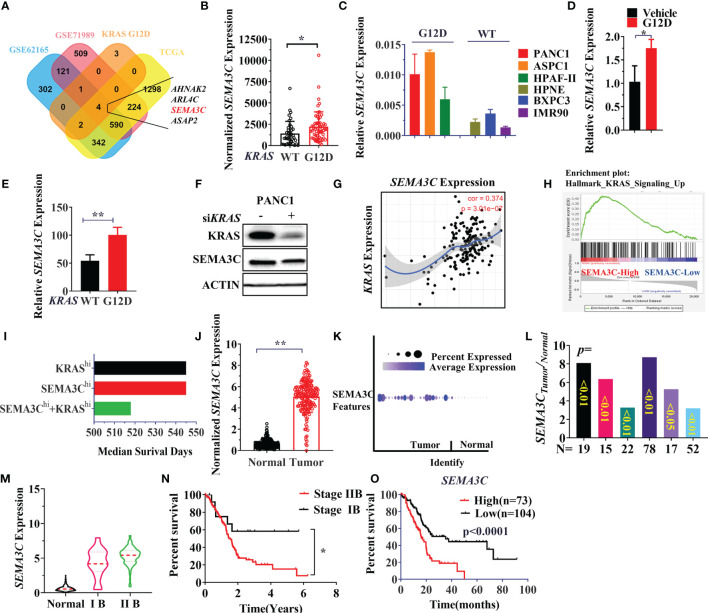
**(A)** Simultaneous highly expressed genes in KRAS G12D mutation patients/cells, TCGA and GEO datasets (GSE62125/GSE71989). The normalized gene expression data were used for the comparison of two groups, the FC (fold change)>=1.5, p-Value<=0.05. **(B)** SEMA3C expression in *KRAS* WT and G12D mutation patients from TCGA data. **(C)** SEMA3C expression in cell lines with or without KRAS G12D mutation evaluated by the qPCR assay (KRAS WT cell lines: HPNE, BxPC3, IMR90; KRAS G12D cell lines: PANC1, ASPC1, HPAF-II). **(D)** SEMA3C expression was detected by qPCR after overexpression of the KRAS G12D for 48 hours. **(E)** SEMA3C expression in KPC cell lines with or without KRAS G12D mutation, data was collected in the GSE63348 database. **(F)** SEMA3C protein expression after depletion of *KRAS* in PANC1 cells. **(G)** The correlation between *KRAS* and SEMA3C expression analyzed by Timer software **(H)** High expressed or low expressed of SEMA3C enriched signaling pathway by the GSEA analysis. **(I)** Survival analysis of the patient group with high expression of SEMA3C, KRAS and both high expressions by the PROGgeneV2. **(J)** The expression data are first log2(TPM+1) transformed for differential analysis and the log2FC is defined as median (Tumor) and median (TCGA normal + GTEx normal), data were collected and analyzed by GEPIA. **(K)** The expression analysis of the SEMA3C from GSA: CRA001160 data (40). **(L)** SEMA3C expression data from the Oncomine in different groups (Segara; Logsdon; Grutzmann; Badea; Iacobuzio Donahue; Pei). (**M)** SEMA3C expression analyzed in different clinical stages of pancreatic cancer from www.aclbi.com. **(N)** The overall survival of patients analyzed in different stages of pancreatic cancer. **(O)** The overall survival of the SEMA3C expression. Data from KM plot, significance vs. cutoff values between lower and upper quartiles of expression. Data are representative of three independent experiments. T-tests or log-rank Test for the measurements between the two groups; *P < 0.05, **P < 0.01.

An alternative method for targeting the KRAS signaling pathway is to discover the vulnerable genes associated with different KRAS mutations. Since patients with the KRAS G12D mutation suffered a shorter survival time, we next explored potential KRAS G12D dependent genes in pancreatic carcinogenesis. The differentially expressed genes (DEGs) were compared between KRAS WT and KRAS G12D pancreatic cancer cell lines and TCGA patients. Aberrantly expressed genes in cancer can be regarded as oncogenes that could promote or support cancer progression. Then we combined KRAS G12D associated DEGs with the genes that are highly expressed in pancreatic cancer patients in other 3 cohorts (TCGA/GSE62125/GSE71989). AHNAK2, ARL4C, ASAP2, SEMA3C were identified as being highly expressed in both KRAS G12D cell lines and pancreatic cancer patients (**Figure 2A**). Next, we chose SEMA3C for further analysis and verified that SEMA3C was highly expressed in both patients (TCGA) and cell lines with *KRAS* mutations (**Figures 2B, C**). Significantly, overexpression of the KRAS G12D in cultured cancer cells was found to increase the SEMA3C expression (**Figure 2D**). To further validate the expression of SEMA3C associated with *KRAS* G12D mutation, we analyzed KPC cell lines with or without KRAS G12D mutation from the GEO database (41). SEMA3C expression was significantly higher in pancreatic cancer cell lines derived from the KRAS G12D mutation mice than that from the WT mice (**Figure 2E**). Significantly, knockdown of the KRAS expression resulted in decreased SEMA3C expression (**Figure 2F**). Higher SEMA3C expression was correlated with KRAS expression and KRAS related signaling pathways (**Figures 2G, H**). Patients with high expression levels of both SEMA3C and KRAS had shorter survival times compared to those with high levels of either alone in pancreatic cancer patients (**Figure 2I**) (42). As expected, SEMA3C was highly expressed in pancreatic cancer, compared to paired normal tissue in three pancreatic cancer cohorts from TCGA/GSA/Oncomine (**Figures 2J–L**). SEMA3C expression is positively correlated with the clinical stages of pancreatic cancers, while patients with advanced stages of pancreatic cancer have a significantly shorter survival rate (**Figures 2M, N**). Consistent with the previous observations, high SEMA3C expression in pancreatic cancer was linked with a shorter survival time based on the analysis of the TCGA database (**Figure 2O**). Similarly, AHNAK2, ARL4C, ASAP2 were all highly expressed in pancreatic cancers, correlated with KRAS G12D mutation and could predict survival in pancreatic cancers (**Figure S3**).

### SEMA3C Knockdown Impairs Cell Growth, Colony Formation, and Cell Cycle Arrest

**Figure 3:**
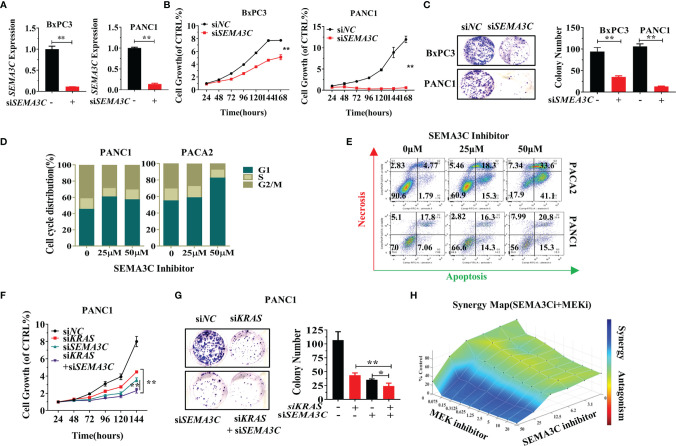
**(A)** Expression levels of SEMA3C detected by qPCR after treatment with *siSEMA3C*. **(B)** The cell growth of pancreatic cells evaluated by the CellTiter-Glo^®^2.0 assay. **(C)** Colony formation assays were assessed after the knockdown of the *SEMA3C* expression in pancreatic cancer cells. **(D)** Cell cycle evaluation in pancreatic cancer cells after treatment of SEMA3C inhibitor 24 hours. **(E)** Cell necrosis and apoptosis evaluation in pancreatic cancer cells after treatment of *SEMA3C* inhibitor 24 hours. **(F, G)** The synergistic effect of the si*KRAS* and si*SEMA3C* in cell growth and cell colony formation assessed after knocking down the expression of KRAS, SEMA3C, or both in PANC1 cells. **(H)** Synergistic effects on cell viability evaluated by combining trametinib and SEMA3C inhibitor in KPC cells calculated with combenefit software (43). Data are representative of three independent experiments. T-tests for the measurements between the two groups; *P < 0.05, **P < 0.01.

We next explored the function of SEMA3C in pancreatic cancer. si*NC* and si*SEMA3C* were transfected into BXPC3 and PANC1 cells and the expression of SEMA3C was measured by RT-PCR to confirm the silencing of endogenous SEMA3C mRNA expression (**Figures 3A**, **S5A**). Knockdown of SEMA3C by siRNA resulted in the impairment of both growth and colony formation in PANC1 cells and BXPC3 cells (**Figures 3B, C**). Inhibition of SEMA3C with the SEMA3C special inhibitor 3,5,4’-Tribromosalicylanilide resulted in cell cycle arrest and apoptosis increase in pancreatic cancer cell lines (**Figures 3D, E**). Simultaneous suppression of KRAS and SEMA3C by siRNA shows a superimposed inhibition effect in cell growth and colony formation (**Figures 3F, G**). Furthermore, the combination of trametinib (MEK inhibitor, downstream of *KRAS)* and SEMA3C inhibitor can induce synergistic effect in KRAS G12D cells calculated by the combenefit software (**Figure 3H**),while no synergy effect in KRAS WT cells(**Figure S5C**).

### SEMA3C Promotes Tumor Cell Survival by Autophagy Induction

**Figure 4:**
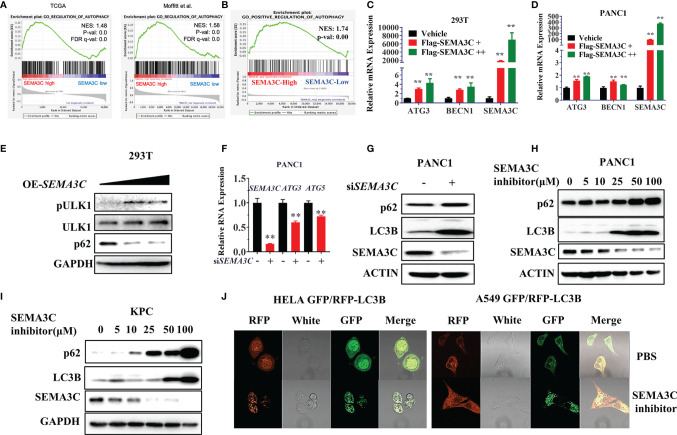
**(A, B)** The correlation between SEMA3C expression and autophagy signaling pathway analyzed with TCGA and CRA001160 data by GSEA software. **(C, D)** Overexpression of the SEMA3C in 293T and PANC1 cells for 48 hours, autophagy induction genes mRNA levels were detected by the qPCR assay. **(E)** Overexpression of SEMA3C in 293T cells for 48 hours, autophagy initial protein ULK1 phosphorylation status and total protein were detected. **(F)** Knockdown of SEMA3C in PANC1 cells induces autophagy initial gene ATG3 and ATG5 mRNA expression. **(G)** Knocking down the SEMA3C in PANC1 cell lines, p62, and LC3B protein levels were detected by western blot. **(H, I)** Dose dependent on p62 and LC3B protein level after treatment by the SEMA3C inhibitors. **(J)** GFP-RFP-LC3B labeled cells were treated by the SEMA3C inhibitor, the LC3B puncta observed by the confocal image. Data are representative of three independent experiments. T-tests for the measurements between the two groups; **P < 0.01.

KRAS mutant tumors have been shown to be dependent on autophagy for growth and survival (44). Next, we investigated whether the SEMA3C expression could mediate autophagy progress. We first evaluated the TCGA data and found that SEMA3C expression shows a strong correlation with the autophagy pathway expression (**Figures 4A, B**). We then overexpressed SEMA3C in 293T and PANC1 cells and found that the RNA expression levels of the autophagy induction genes ATG3 and Beclin1 were upregulated, which represented the initiation of autophagy (**Figures 4C, D** and **S5B**). Moreover, overexpression of SEMA3C in 293T cells showed autophagy induction by evaluating ULK1 phosphorylation levels and p62 protein expression (**Figure 4E**). Knockdown of SEMA3C resulted in a decrease of ATG3 and ATG5 in the mRNA level (**Figure 4F**). Knockdown of SEMA3C resulted in a significant accumulation of LC3B and P62 protein (**Figure 4G**), which indicated autophagy was inhibited based on previous reports that indicate that these proteins accumulate when autophagy is disrupted (45, 46). Furthermore, the upregulation of LC3B and P62 was cumulative and dose-dependent in different pancreatic cells (**Figures 4H, I**). Stably expressing GFP/RFP-LC3B cells are commonly used for evaluating autophagy flux progress representing the autophagy induction or inhibition (47), and we found that treating these cells with SEMA3C inhibitors increased more yellow puncta which meant the autophagy process was blocked (**Figure 4J**).

### SEMA3C Leads to an Immunosuppressive Environment by Upregulation of PD-L1 and Macrophage Polarization

**Figure 5:**
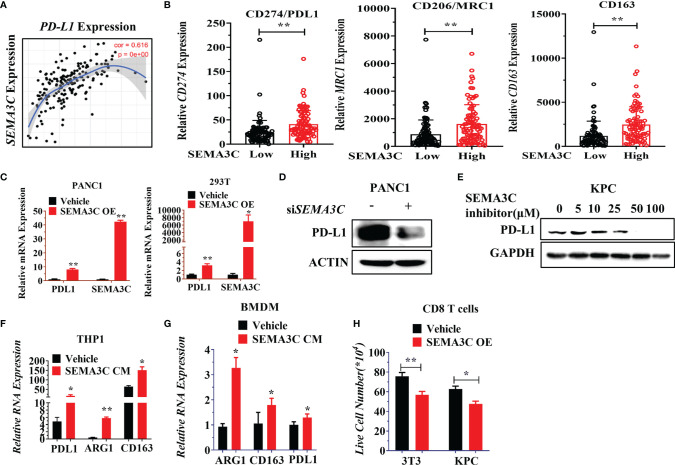
**(A)** The correlation between SEMA3C and PD-L1 analyzed by Timer software. **(B)** PD-L1, MRC1, *CD163* expression in either SEMA3C high expression or low expression groups. The groups were divided into two groups by the mean of SEMA3C in the TCGA database. **(C)** PD-L1 mRNA expression detected after the overexpression of SEMA3C in different cell lines. **(D)** PD-L1 protein expression was detected after knocking down the SEMA3C expression. **(E)** PD-L1 protein expression was detected after treatment of different doses of SEMA3C inhibitor in the KPC cell line for 24 hours. **(F)** Expression of PD*-*L1, ARG1, and CD163 were detected by qPCR in THP1 cells after being treated by the conditional medium derived from either PANC1 cells or PANC1 cells overexpressing SEMA3C. **(G)** Expression of PD-L1, ARG1, and CD163 were detected by qPCR in BMDM cells after being treated by the conditional medium derived from KPC cells or KPC cells overexpressing SEMA3C. **(H)** Activated CD8+ T cells co-cultured with normal 3T3 and KPC cells or 3T3 and KPC cells with overexpressed SEMA3C for 24 hours before the suspended T cells number were counted. The error bars indicate the SD of three independent experiments: *P < 0.05, **P < 0.01.

Preclinical reports have shown evidence linking oncogenic KRAS mutations and PD-L1 expression in cancers, which reduces the tumor-specific T cell’s function in the tumor microenvironment (TME) (48). KRAS up-regulation of PD-L1 has further been shown to occur through ERK signaling pathways (49). Interestingly, SEMA3C knockdown also could control the ERK signaling pathway (18), which led us to inquire whether SEMA3C could mediate or be involved in the regulation of PD-L1 expression in tumor and tumor associated macrophages (TAMs). NRP1, one of the receptors of SEMA3C, was highly expressed on TAMs. NRP1 on macrophages triggers VEGFR1 activation and migration of macrophages to become TAMs (50). We checked the expression of the NRP1 in different cell types in pancreatic cancer (51). NRP1 are not only expressed in pancreatic cancer but also in macrophages/monocytes, this suggests that SEMA3C could affect the macrophage polarization by binding the NRP1 expressed in macrophages (**Figure S4**). We then examined the correlated expression of both *PD-L1* and SEMA3C in pancreatic cancer with TCGA data by Timer software (**Figure 5A**). We found that immunosuppressive genes (M2 makers: CD274, CD206, CD163) which are mainly expressed in macrophages were higher in the SEMA3C^high^ group (**Figure 5B**). To confirm that SEMA3C expression could affect the PD-L1 expression, we overexpressed SEMA3C in PANC1 and 293T cell lines, and observed upregulated PD-L1 in these two cell lines after SEMA3C overexpression (**Figure 5C**). Knocking down SEMA3C led to PD-L1 decrease (**Figure 5D**). Additionally, treatment with a SEMA3C inhibitor significantly decreased the PD-L1 expression in KPC cells in a dose dependent manner (**Figure 5E**). Since SEMA3C is a secreted glycoprotein, tumor secreted SEMA3C may impact host myeloid/macrophage PD-L1 expression or polarization through modulation of the tumor-suppressive environment (52). THP1 and BMDM (Bone Marrow Derived Macrophage) were used as myeloid/macrophage models to test the PD-L1 expression or polarization *in vitro*. These cells were treated by the conditional medium collected from the PANC1 or KPC cells with or without the SEMA3C overexpression and we observed an increase in the PD-L1, CD163 or ARG1 mRNA levels (**Figures 5F, G**). Moreover, PD-L1 high expression can mediate T cell anergy by stimulating the PD1/PD-L1 signaling pathway, which could lead to the T cell death (53). To test this, we co-cultured activated T cells with either 3T3 or KPC vehicle cells, or 3T3 or KPC cell overexpressed SEMA3C. We found that the SEMA3C high expression groups showed a significant decrease in live T cells relative to cells with normal SEMA3C expression (**Figure 5H**).

### SEMA3C Inhibitor Retarded Tumor Growth *In Vivo* Pancreatic Cancer Model

**Figure 6:**
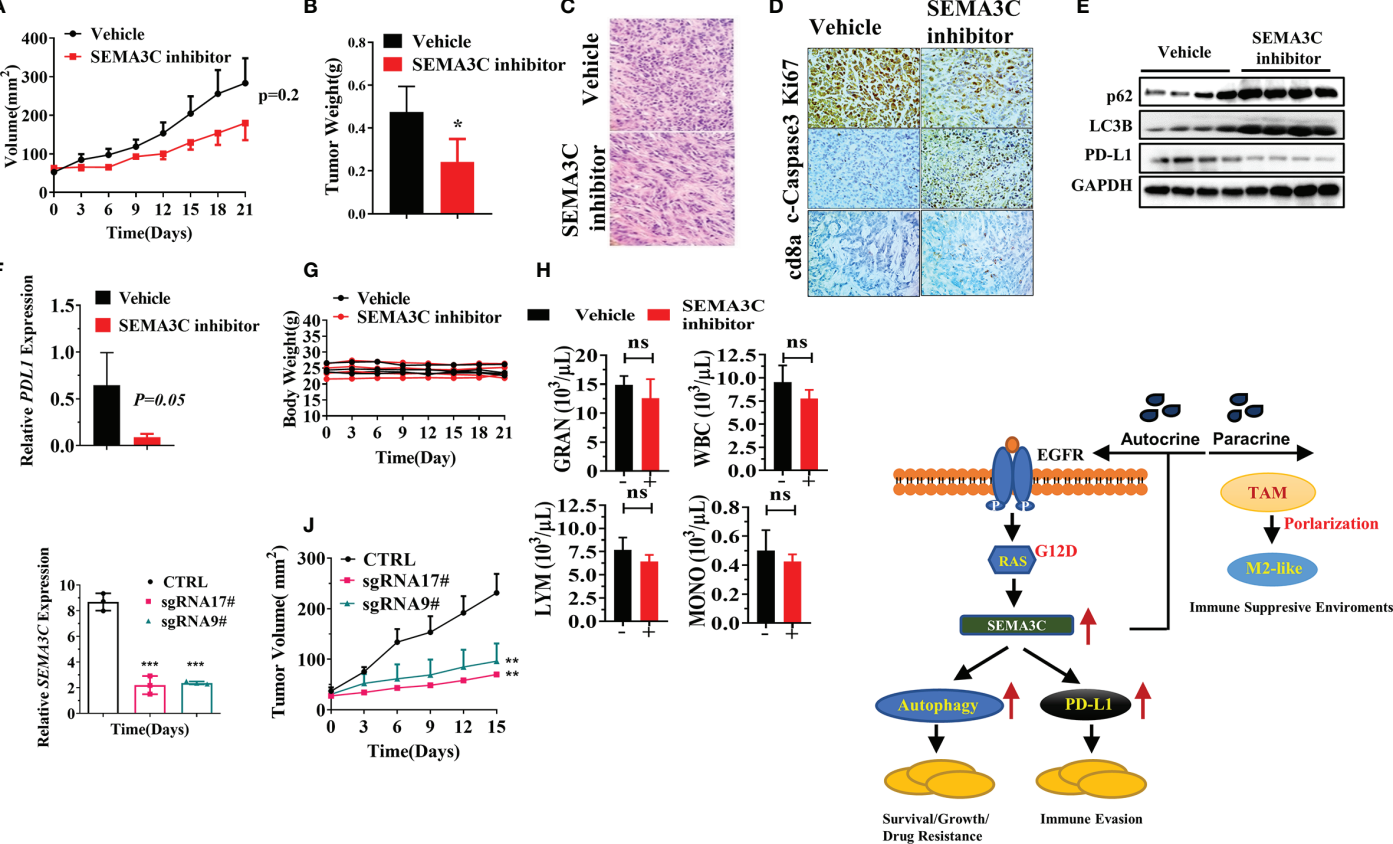
**(A)** The tumor volume of the KPC tumor bearing mice model treated with SEMA3C inhibitor (20 mg/kg) or vehicle for 7 doses (n=5). The tumor length and width were measured every 3 days, and the tumor volume was calculated by the formula=1/2 length*width^^2^. **(B)** The weight of the tumors collected after euthanizing the mice treated with either the vehicle or SEMA3C inhibitor group. **(C)** H&E histology was evaluated in the vehicle and inhibitor groups. **(D)** Ki67 cleaved-Caspase3 and CD8a were evaluated in the vehicle and inhibitor groups. **(E)** Total proteins were extracted from the tumor, and autophagy related proteins p62, LC3B, and PD-L1 were detected by the western blot. **(F)** PD-L1 mRNA expression was detected by qPCR in tumors of KPC-Luc bearing mice. **(G)** The body weight of the Vehicle and SEMA3C inhibitor group were recorded every three days. **(H)** The complete blood counts were detected 48 hours after the inhibitor injection (HemaTrue, Loveland, Co). **(I)** SEMA3C expression were evaluated by qPCR in Vehicle, SEMA3C sgRNA 9# and 17#. **(J)** The tumor volume of the Vehicle, SEMA3C sgRNA 9# and 17# KPC cells subcutaneous model, the tumor length and width were measured every 3 days, and the tumor volume was calculated by the formula=1/2 length*width^^2^. **(K)** Schematic illustration of probable mechanism of SEMA3C role in pancreatic cancer. Data are representative of three independent experiments. T-tests for the measurements between the two groups; *P < 0.05, **P < 0.01, ***P < 0.001, ns, no significance.

To explore the role of SEMA3C in pancreatic cancer *in vivo*, we created a KPC tumor model by subcutaneously injecting 5×10^5^ mouse KPC-Luc cells into C57/BL6 mice (Charles River). After the tumors became established, mice were treated with the vehicle or SEMA3C inhibitor (20 mg/kg every other day for a total of 7 doses). We found that the tumor volume and weight in the SEMA3C inhibition group were significantly smaller than that in the vehicle group (**Figures 6A, B**). H&E results showed that the tumor was dense in the vehicle group while there were fewer tumor cells and more stroma in the group treated with the SEMA3C inhibitor (**Figure 6C**). The expression of Ki67 is strongly associated with tumor cell proliferation and growth and our IHC results show that the level of Ki67 is much higher in the vehicle group than that in the SEMA3C inhibitor group. On the contrary, the levels of cleaved caspase 3, a common cell apoptosis marker, is much lower in the vehicle group. We also detected that the CD8a positive staining was much greater in the SEMA3C inhibitor treated group (**Figure 6D**). Together, these results indicated that the tumorigenesis of the KPC tumor bearing mice was inhibited by SEMA3C inhibition. We further explored the role of SEMA3C in regulating the autophagy process and PD-L1 expression in the tumors and found that the SEMA3C inhibitor can influence the expression levels of these proteins’ expression *in vivo* (**Figure 6E**). The enhanced p62 and LC3B expression imply that the autophagy process was blocked by the SEMA3C inhibitor (**Figure 6E**). Meanwhile, PD-L1 expression was decreased after the treatment (**Figure 6E**). PD-L1 mRNA level was also decreased in the SEMA3C inhibitor treated group (**Figure 6F**). Importantly, the SEMA3C inhibitor had no observed toxicity in mice by measuring the body weight and the complete blood counts (**Figures 6G, H**). Knock down the SEMA3C expression by CRISPR-Cas9 in KPC cells and the xenograft model shows that SEMA3C impairs the growth *in vivo* (**Figures 6I, J**). These findings demonstrated that SEMA3C inhibition suppresses tumor growth *in vivo* by inhibiting cancer cell growth and altering the immune response within the tumor microenvironment. The Schematic illustration of probable mechanism of SEMA3C role in pancreatic cancer (**Figure 6K**).

## Discussion

There is currently no effective therapy that can cure pancreatic cancer. KRAS mutations occur in about 90% of pancreatic cancer and KRAS is a significant oncogene for the initiation of this cancer type. There are several types of KRAS mutations including the G12D, G12C, G12V, and G12R in pancreatic cancers which may play different roles. KRAS has been generally undruggable until the recent discovery of Sotorasib (AMG510, a specific covalent inhibitor of KRAS G12C mutation)), which has shown excellent therapeutic prospects. However, different KRAS mutations play different roles by allele-specific signaling pathways and the patient’s survival time also varies from different mutation types. The KRAS G12D mutation is frequent in pancreatic cancer which makes targeting KRAS with the G12D mutation more prospective than the other mutations in pancreatic cancer therapy.

Autophagy is a cell survival mechanism induced by various conditions such as drug treatment, hypoxia or gene mutation, which helps cancer cells survive and induces drug resistance (54). High basal autophagy rates have been described in several human pancreatic ductal adenocarcinoma (PDAC) cell lines and autophagy is upregulated in the latter stages of pancreatic ductal neoplasia progressing to PDAC (55). Autophagy is elevated in KRAS-driven cancers but not normal tissue and is essential for tumor growth (56). Acute KRAS inhibition by the downstream effector increases rather than decreases autophagy. In addition, the ERK inhibition phenotype inhibits KRAS and stimulates increased autophagy flux. Inhibition of KRAS-RAF-MEK-ERK signaling triggers autophagy which could protect PDAC cells from the cytotoxic effects of KRAS pathway inhibition (57, 58).

Pancreatic cancer is known to have a highly immunosuppressive microenvironment (59). The immunosuppressive microenvironment allows tumor cells to escape immune surveillance and elimination of the anti-tumor immune system (60). One of the intrinsic determinants of immunogenicity is the expression of PD-L1 in tumors or tumor-related cells within the tumor microenvironments. Different signaling pathways change can induce the production of constitutive PD-L1 (61). PD-L1 is expressed not only in tumor cells (62) but also highly expressed in tumor-associated macrophages, which are the most abundant immune cell populations in the tumor microenvironment (63).

By multiple-genomics data mining we found that the KRAS G12D mutation was significantly associated with the survival of pancreatic patients and identified 4 genes that are associated with KRAS G12D mutation and highly expressed in pancreatic cancer and could regarded as promising targets for the pancreatic cancer therapy. Interestingly, very recent reports also reach a similar conclusion for these genes. For example, ASAP2 was identified as the novel driver gene and potential druggable target in pancreatic cancer (64). AHNAK2 was identified as differentially expressed proteins in KRAS mutants compared to cells lacking Ras pathway mutation (65). ARL4C was identified as RAF1-MEK/ERK pathway-dependent gene in ameloblastoma cell proliferation and osteoclast formation and as down localization of KRAS in pancreatic cancer (66, 67). Here we explored the SEMA3C role in pancreatic cancer. SEMA3C was highly expressed in pancreatic cancers, especially in cells and patients with high KRAS G12D expression, and high SEMA3C expression was enriched in the signaling pathways upregulated by KRAS. Both KRAS and SEMA3C high expression genotypes shortened the survival time in pancreatic cancer patients. SEMA3C played a significant role in cell growth, cell colony formation, cell cycle arrest, and the increased apoptosis or necrosis of pancreatic cancer cells. Furthermore, a double knockdown of KRAS and SEMA3C shows significant synergistic inhibition of PANC1 cell growth and colony formation. We also demonstrated the synergistic effect in killing pancreatic cancer cells with the combination of MEK inhibitor and SEMA3C inhibitor. The process of autophagy is highly active in pancreatic cancer cells and autophagy is upregulated in KRAS mutated cells. Autophagy clears the dying organelles in damaged cancer cells to create the needed energy to survive and divide (68). The role of SEMA3C expression on autophagy regulation and immune modulation of TME has not been reported before. Herein, we found that high SEMA3C expression has enriched the autophagy process and SEMA3C played an active role in promoting autophagy. Next, we found that SEMA3C overexpression or inhibition can significantly regulate the expression of the autophagy-related markers in pancreatic cancer lines. Furthermore, SEMA3C expression can increase the PD-L1 expression in both tumor and immune cells which may mediate the immunosuppressive environment in pancreatic cancer. A SEMA3C inhibitor showed a significant inhibition effect on the growth of KPC-Luc bearing tumors *in vivo*. We further demonstrated the dramatic autophagy inhibition response by exploring the autophagy-related proteins and significant decrease in PD-L1 level in tumor tissue with treatment of SEMA3C inhibitor.

Overall, SEMA3C expression is associated with KRAS G12D mutation, which is highly expressed in pancreatic patients. Knocking down or inhibiting SEMA3C shows the tumor inhibition *in vitro* or *in vivo*. SEMA3C affects the regulation of autophagy and PD-L1. The study indicates that SEMA3C is a potentially promising and attractive target for pancreatic cancer therapy especially in patients with a G12D mutation in KRAS.

## Data Availability Statement

The raw data supporting the conclusions of this article will be made available by the authors, without undue reservation.

## Ethics Statement

The animal study was reviewed and approved by The Animal Use and Care Administrative Advisory Committee of the University of California, Davis (IACUC #20265).

## Author Contributions

DZ and YL contributed to conception and design of the study. DZ collected the data and organized the database. DZ performed the statistical analysis. DZ wrote the first draft of the manuscript. DZ, AL, EK, C-IH, MH, TL and YL revise section of the manuscript. All authors contributed to manuscript revision, read, and approved the submitted version.

## Conflict of Interest

The authors declare that the research was conducted in the absence of any commercial or financial relationships that could be construed as a potential conflict of interest.

## Publisher’s Note

All claims expressed in this article are solely those of the authors and do not necessarily represent those of their affiliated organizations, or those of the publisher, the editors and the reviewers. Any product that may be evaluated in this article, or claim that may be made by its manufacturer, is not guaranteed or endorsed by the publisher.
