# Supplementary material for: SEMA3C Supports Pancreatic Cancer Progression by Regulating the Autophagy Process and Tumor Immune Microenvironment
Source: Front Oncol. 2022 Jun 16;12:890154. doi: 10.3389/fonc.2022.890154 (PMC9243227; doi:10.3389/fonc.2022.890154)
Supplement: Supplementary file 1 [file DataSheet_1.pdf]

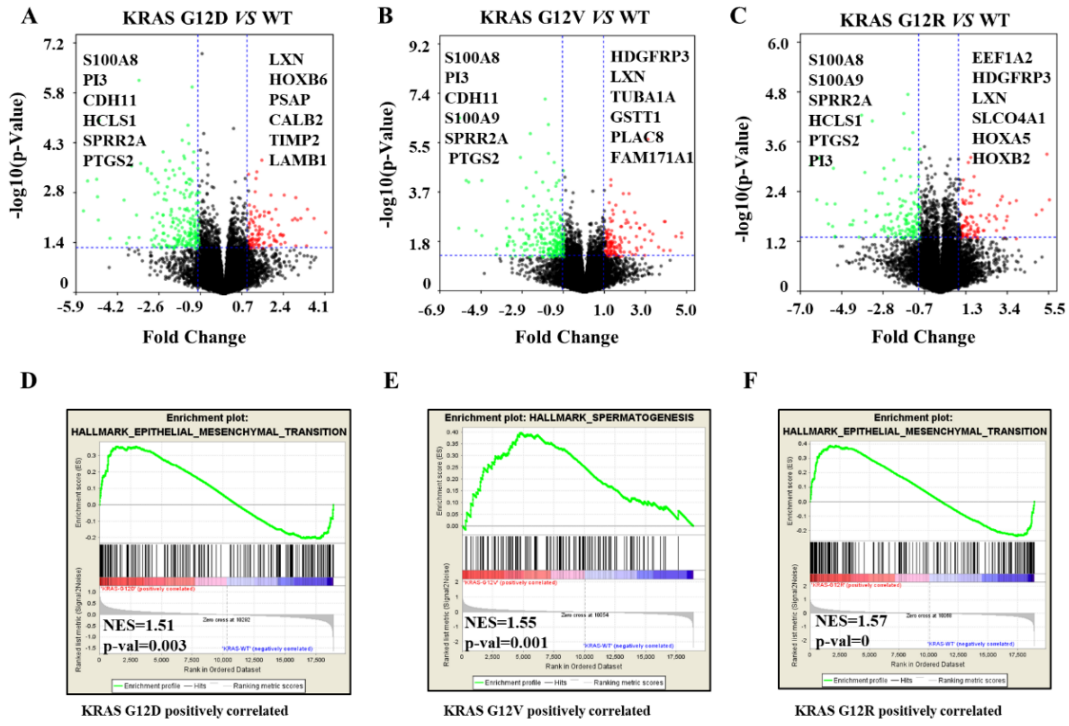

Figure S1 (A-C) Differential genes expression in different *KRAS* mutation subtype cell lines. (**D-F**) Major signaling pathway enrichment in different *KRAS* mutation subtype cell lines. The gene expression profiles were obtained from the Cell Line Encyclopedia.

CELL LINES	**CHR**	**CDS mut**	**AA mut**
SNU410_PANCREAS	12	c.35G>A	p.G12D
ASPC1_PANCREAS	12	c.35G>A	p.G12D
L33_PANCREAS	12	c.35G>A	p.G12D
PANC1005_PANCREAS	12	c.35G>A	p.G12D
PL45_PANCREAS	12	c.35G>A	p.G12D
BXPC3_PANCREAS	12	NO	NO
TCCPAN2_PANCREAS	12	c.34G>C	p.G12R
KCIMOH1_PANCREAS	12	c.35G>A	p.G12D
HPAFII_PANCREAS	12	c.35G>A	p.G12D
CAPAN2_PANCREAS	12	c.35G>T	p.G12V
DANG_PANCREAS	12	c.35G>T	p.G12V
PANC0327_PANCREAS	12	c.35G>T	p.G12V
CFPAC1_PANCREAS	12	c.35G>T	p.G12V
KLM1_PANCREAS	12	c.35G>A	p.G12D
PANC0203_PANCREAS	12	c.35G>A	p.G12D
CAPAN1_PANCREAS	12	c.35G>T	p.G12V
PATU8902_PANCREAS	12	c.35G>T	p.G12V
PK1_PANCREAS	12	c.35G>A	p.G12D
PANC0504_PANCREAS	12	c.35G>A	p.G12D
PK59_PANCREAS	12	c.35G>A	p.G12D
SU8686_PANCREAS	12	c.35G>A	p.G12D

SUIT2_PANCREAS	12	c.35G>A	p.G12D
PANC0813_PANCREAS	12	c.35G>A	p.G12D
KP2_PANCREAS	12	c.34G>C	p.G12R
HPAC_PANCREAS	12	c.35G>A	p.G12D
SNU324_PANCREAS	12	NO	NO
SW1990_PANCREAS	12	c.35G>A	p.G12D
HUPT3_PANCREAS	12	c.34G>C	p.G12R
SNU213_PANCREAS	12	c.35G>T	p.G12V
YAPC_PANCREAS	12	c.35G>T	p.G12V
KP3_PANCREAS	12	c.35G>T	p.G12V
PSN1_PANCREAS	12	c.34G>C	p.G12R
PANC0403_PANCREAS	12	c.35G>A	p.G12D
PK45H_PANCREAS	12	c.35G>A	p.G12D
HUPT4_PANCREAS	12	c.35G>T	p.G12V
QGP1_PANCREAS	12	c.35G>T	p.G12V
PANC1_PANCREAS	12	c.35G>A	p.G12D
MIAPACA2_PANCREAS	12	c.35G>T	p.G12C
PATU8988S_PANCREAS	12	c.35G>T	p.G12V
KP4_PANCREAS	12	c.35G>A	p.G12D
PATU8988T_PANCREAS	12	c.35G>T	p.G12V

Table 1 Different *KRAS* mutation types in pancreatic cancer cells.

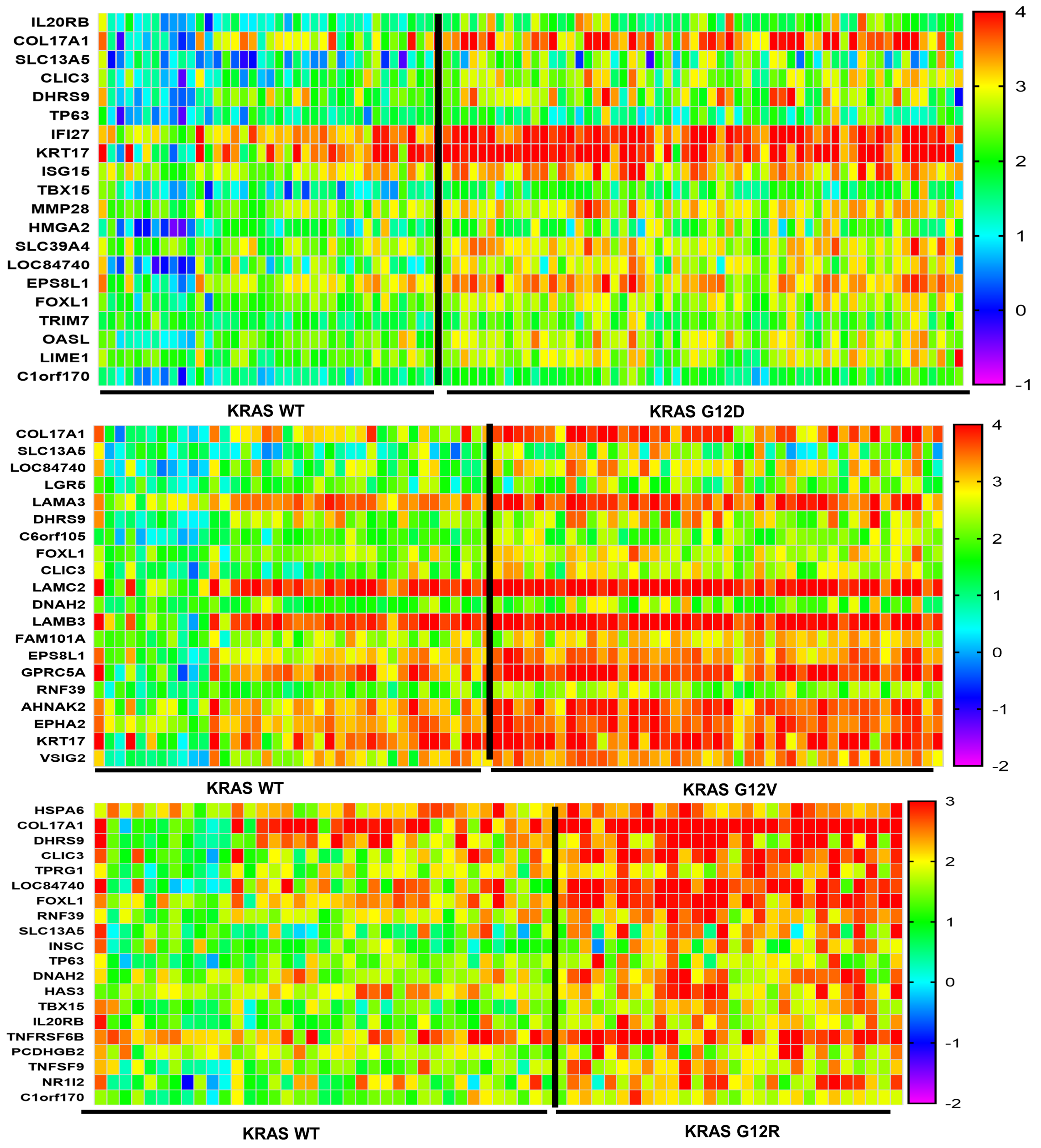

Figure S2 Different expressed genes (FC>=1.5, p Value<=0.05) between KRAS WT and KRAS mutations. Data was collected from TCGA.

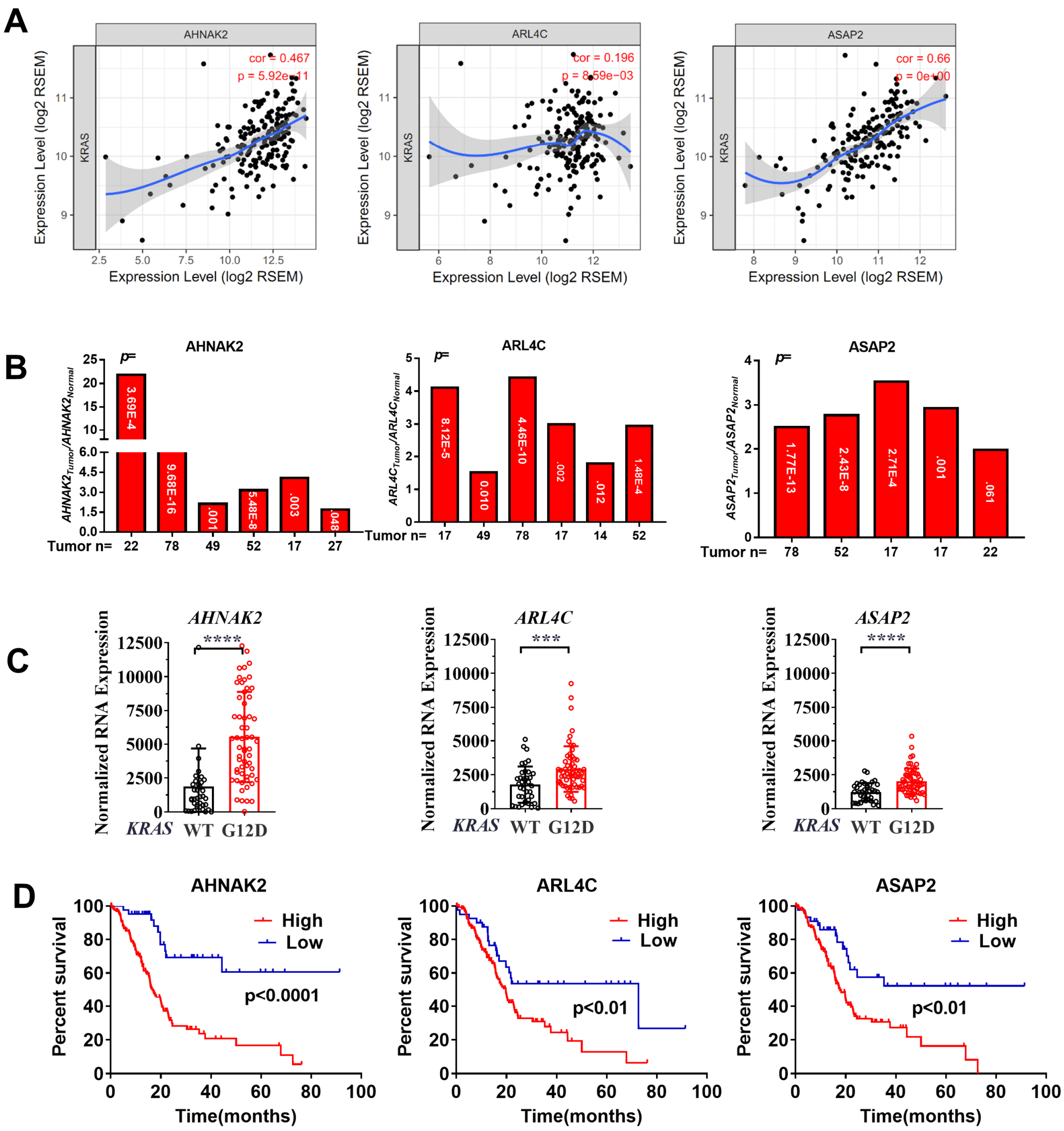

Figure S3 (A-D) Evaluation of the AHNAK2/ARL4C/ASAP2 in pancreatic cancer patients for correlation with KRAS expression(**A**), expression in Tumor/Normal (**B**), expression in G12D/WT**(C)** and clinical outcome**(D).** T-tests or log-rank Test for the measurements between the two groups *P < 0.05, **P < 0.01.

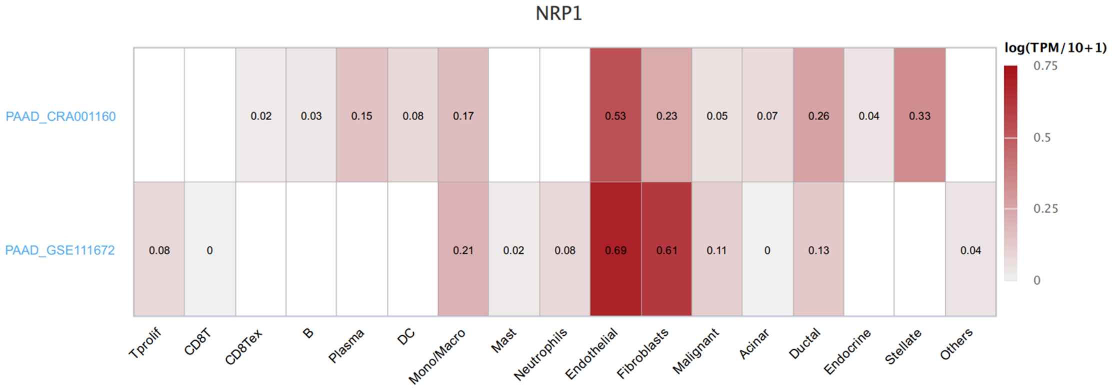

Figure S4 NRP1 expression in different cell types in pancreatic cancer, data were collected form TISCH[51].

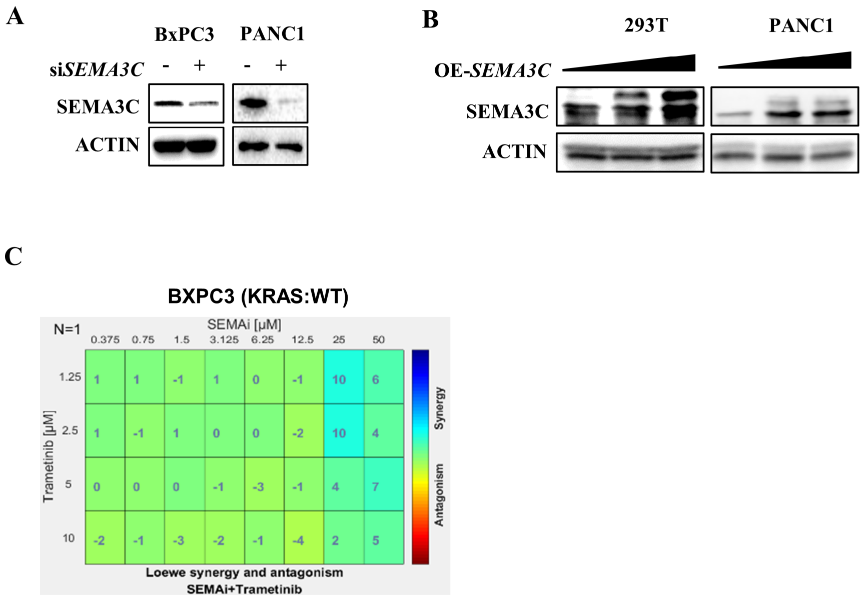

Figure S5

The protein level detected by WB for knocking down **(A)** or overexpression of SEMA3C**(B).**

(C) Synergistic effects on cell viability evaluated by combining trametinib and SEMA3C inhibitor in BXPC3 cells calculated with combenefit software.

Primer's list (Designed from Primer Bank: https://pga.mgh.harvard.edu/primerbank/index.html):

**Mouse:**	**Primer**
ARG1-F	5-CTCCAAGCCAAAGTCCTTAGAG-3
ARG1-R	5-AGGAGCTGTCATTAGGGACATC-3
CD163-F	5- TCCACACGTCCAGAACAGTC-3

CD163-R	5- CCTTGGAAACAGAGACAGGC-3
PD-L1-F	5-GCTCCAAAGGACTTGTACGTG-3
PD-L1-R	5-TGATCTGAAGGGCAGCATTTC-3
GAPDH-F	5-TGGATTTGGACGCATTGGTC-3
GAPDH-R	5-TTTGCACTGGTACGTGTTGAT-3
**Human:**	**Primer**
ARG1-F:	5- GTGGAAACTTGCATGGACAAC-3
ARG1-R	5- AATCCTGGCACATCGGGAATC-3
PD-L1-F	5- TGGCATTTGCTGAACGCATTT-3
PD-L1-R	5- TGCAGCCAGGTCTAATTGTTTT-3
CD163-F	5- TTTGTCAACTTGAGTCCCTTCAC-3
CD163-R	5- TCCCGCTACACTTGTTTTCAC-3
SEMA3C-F	5- TTTGCGTGTTGGTTGGAGTAT-3
SEMA3C-R	5- TCCTGTAGTCTAAAGGATGGTGG-3
ATG3-F:	5- GACCCCGGTCCTCAAGGAA-3
ATG3-R	5- TGTAGCCCATTGCCATGTTGG-3
ARG5-F	5- AGAAGCTGTTTCGTCCTGTGG-3
ARG5-R	5- AGGTGTTTCCAACATTGGCTC-3
GAPDH-F	5- GGAGCGAGATCCCTCCAAAAT-3
GAPDH-R	5- GGCTGTTGTCATACTTCTCATGG-3
